# Towards a Humanized Mouse Model of Liver Stage Malaria Using Ectopic Artificial Livers

**DOI:** 10.1038/srep45424

**Published:** 2017-03-31

**Authors:** Shengyong Ng, Sandra March, Ani Galstian, Nil Gural, Kelly R. Stevens, Maria M. Mota, Sangeeta N. Bhatia

**Affiliations:** 1 Department of Biological Engineering, Massachusetts Institute of Technology, Cambridge, MA, USA; 2 Health Sciences and Technology/Institute for Medical Engineering and Science, Massachusetts Institute of Technology, Cambridge, MA, USA; 3 Broad Institute, Cambridge, MA, USA; 4 Unidade de Malária, Instituto de Medicina Molecular, Universidade de Lisboa, 1649-028 Lisboa, Portugal; 5 Howard Hughes Medical Institute, Koch Institute, and Electrical Engineering and Computer Science, Massachusetts Institute of Technology, Cambridge, MA, USA.; 6 Department of Medicine, Brigham and Women’s Hospital, Boston, MA, USA

## Abstract

The malaria liver stage is an attractive target for antimalarial development, and preclinical malaria models are essential for testing such candidates. Given ethical concerns and costs associated with non‐human primate models, humanized mouse models containing chimeric human livers offer a valuable alternative as small animal models of liver stage human malaria. The best available human liver chimeric mice rely on cellular transplantation into mice with genetically engineered liver injury, but these systems involve a long and variable humanization process, are expensive, and require the use of breeding-challenged mouse strains which are not widely accessible. We previously incorporated primary human hepatocytes into engineered polyethylene glycol (PEG)-based nanoporous human ectopic artificial livers (HEALs), implanted them in mice without liver injury, and rapidly generated human liver chimeric mice in a reproducible and scalable fashion. By re-designing the PEG scaffold to be macroporous, we demonstrate the facile fabrication of implantable porous HEALs that support liver stage human malaria (*P. falciparum*) infection *in vitro*, and also after implantation in mice with normal liver function, 60% of the time. This proof-of-concept study demonstrates the feasibility of applying a tissue engineering strategy towards the development of scalable preclinical models of liver stage malaria infection for future applications.

Malaria affects 250 million people and causes more than half a million deaths each year^1^. As an obligatory stage of the malaria life cycle that occurs soon after infection of the human host, the *Plasmodium* liver stage is an attractive target for the development of antimalarial drugs and vaccines, especially with the current goal of malaria eradication^2^. Recent advances in *in vitro* models of liver stage malaria in primary human hepatocytes have increased the prospects of rapidly identifying potential drugs with activity against the liver stages^3^,^4^. However, preclinical *in vivo* models for liver stage malaria are not widely available^5^, and such models are needed to prioritize leads from *in vitro* antimalarial phenotypic drug screens. Due to species-specific differences in host hepatic drug metabolism^6^, *Plasmodium* species-specific differences in host tropism^7^ and drug resistance mechanisms^8^, and a preference for small animal models over non-human primate models due to ethical and logistical issues, humanized mouse models have emerged as an important route for the preclinical testing of potential antimalarial drugs^5^,^9^,^10^,^11^.

Humanized mouse models containing a human liver component have been generated using both genetic engineering and xenotransplantation approaches^9^,^12^. These models typically involve the transplantation of primary human hepatocytes into genetically engineered immunocompromised mice that exhibit either spontaneous liver injury due to urokinase plasminogen activator overexpression (uPA^+/+^/SCID)^13^, or liver injury due to an inducible FAH disruption (FAH^−/−^)^14^ or inducible expression of herpes simplex virus thymidine kinase (TK-NOG)^15^ or FK508-caspase 9 fusion protein (AFC8)^16^. Although uPA^+/+^/SCID, FAH^−/−^ and TK-NOG mice are infectible with *P. falciparum, P. vivax* and *P. ovale*^17^,^18^,^19^,^20^,^21^, genetic liver injury models are characterized by long engraftment times on the order of months, low and variable engraftment efficiencies, ongoing mouse liver injury during human hepatic reconstitution, and the use of breeding-challenged, costly mouse strains that are not widely accessible^9^,^12^. In contrast, a recently described approach circumvents some of these issues by implanting human ectopic artificial livers (HEALs) in mice without host liver injury in a low-cost, rapid, and scalable fashion^22^. These poly(ethylene glycol) (PEG)-based HEALs leveraged classical hepatic tissue engineering techniques to stabilize primary human hepatocyte function prior to and after implantation, such as introducing an appropriate supportive coculture cell type^23^,^24^ and promoting three-dimensional (3D) hepatic spheroid formation^25^,^26^,^27^,^28^. As a result, tissue-engineered HEALs not only sustained primary human hepatocyte survival and function in three dimensions (3D), but also engrafted after ectopic implantation *in vivo*, allowing the facile modeling of human-specific drug metabolism in a mouse^22^. However, first-generation HEALs were fabricated using a polymer with nanometer pore sizes and thus may not support infection by micron-sized hepatotropic pathogens such as *Plasmodium* sporozoites.

In this study, we sought to establish an implantable model of liver stage *Plasmodium* infection of primary human hepatocytes in PEG-based HEALs. We first redesigned the biomaterial scaffold to support sporozoite entry by synthesizing macroporous PEG cryogels. In this system, PEG macromers undergo controlled freezing such that the ice crystals that form during gelation can be subsequently sublimated, leaving a highly porous structure with micron-sized pores^29^. Next, we optimized the functional maintenance of primary human hepatocytes encapsulated in PEG cryogel-based porous human ectopic artificial livers (p-HEALs), tested the feasibility of establishing liver stage *Plasmodium* infection in p-HEALs with three *Plasmodium* species, and characterized the sensitivity of infected p-HEALs to a known antimalarial, primaquine. Finally, we implanted p-HEALs in the intraperitoneal (IP) space of nude mice and demonstrated p-HEAL infection with three *Plasmodium* species *in vivo* in the absence of host liver injury. Together, these data demonstrate the feasibility of creating a humanized mouse model of liver stage human malaria using tissue engineering rather than genetic approaches. This strategy may offer an efficient and scalable method to establish preclinical models of liver stage human malaria.

## Results

### Hepatocytes encapsulated in PEG cryogels are infectible by *Plasmodium* sporozoites

**Figure 1:**
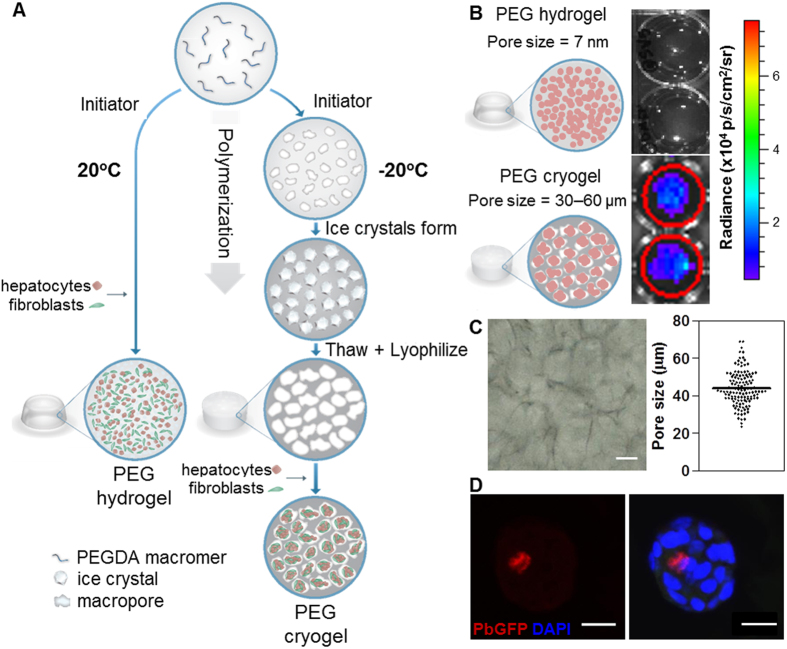
Fabrication of PEG cryogels and characterization of *Plasmodium* sporozoite accessibility to encapsulated cells. (**A**) Schematic of the synthesis of and the encapsulation of hepatocytes and fibroblasts in PEG hydrogels or PEG cryogels. (**B**) Huh7.5 human hepatoma cells (red circles) that were encapsulated in either PEG hydrogels or PEG cryogels were exposed to Pb-GFP-luc sporozoites and bioluminescence imaging was performed 48 h post-exposure. (**C**) Representative bright-field image of a PEG cryogel, and quantification of the cryogel pore size distribution. (**D**) Representative immunofluorescence staining of a GFP-expressing Pb-GFP-luc exoerythrocytic form (EEF), which arose from a Pb-GFP-luc sporozoite that invaded a Huh7.5 cell in a cellular aggregate 48 h post-exposure. Blue DAPI staining marks Huh7.5 nuclei, spread through a PEG cryogel macropore. Scale bars: 25 μm.

Due to the nanoporosity^30^ and non-biodegradable nature of the PEGDA hydrogels that were previously used in PEG-based HEALs^22^ (Fig. 1A), we reasoned that micron-sized *Plasmodium* sporozoites may not be able to access hepatocytes encapsulated within the hydrogel. Since rodent *Plasmodium* sporozoites infect primary human hepatocytes *in vitro*^31^, and due to greater accessibility to rodent compared to human *Plasmodium* sporozoites, initial experiments were performed with a reporter strain of rodent *Plasmodium berghei* (Pb) that expresses both green fluorescent protein (GFP) and firefly luciferase (Pb-GFP-luc)^32^, enabling a live infection read-out by IVIS bioluminescence imaging (BLI). Indeed, when human hepatoma Huh7.5 cells, which are highly infectible with Pb sporozoites^33^, were encapsulated in PEGDA hydrogels and exposed to Pb-GFP-luc sporozoites^32^, no BLI signal was observed at 48 h post-exposure (Fig. 1B).

To improve sporozoite access to hepatocytes encapsulated within PEGDA-based hydrogels, a cryogelation protocol was adapted to introduce macroporosity into PEGDA hydrogels^29^ (Fig. 1A). This procedure included the gelation of PEGDA using chemical redox initiators, ammonium persulfate and TEMED, while the gelation mixture was incubated at −20 °C, and resulted in macroporous PEG cryogels with pore sizes ranging from 30–60 μm (Fig. 1C). When Huh7.5 cells were seeded onto lyophilized PEG cryogels and then exposed to Pb-GFP-luc sporozoites, robust BLI signal was observed at 48 h post-exposure (Fig. 1B), and Pb-GFP-luc liver stage parasites, called exoerythrocytic forms (EEFs), could be detected with an immunofluorescent anti-GFP antibody at this same time point (Fig. 1D). These data imply that Pb-GFP-luc sporozoites can access and infect human hepatoma cells encapsulated within macroporous PEG cryogels.

### PEG cryogel-based human ectopic artificial livers (p-HEALs) maintain primary human hepatocyte phenotypes

**Figure 2:**
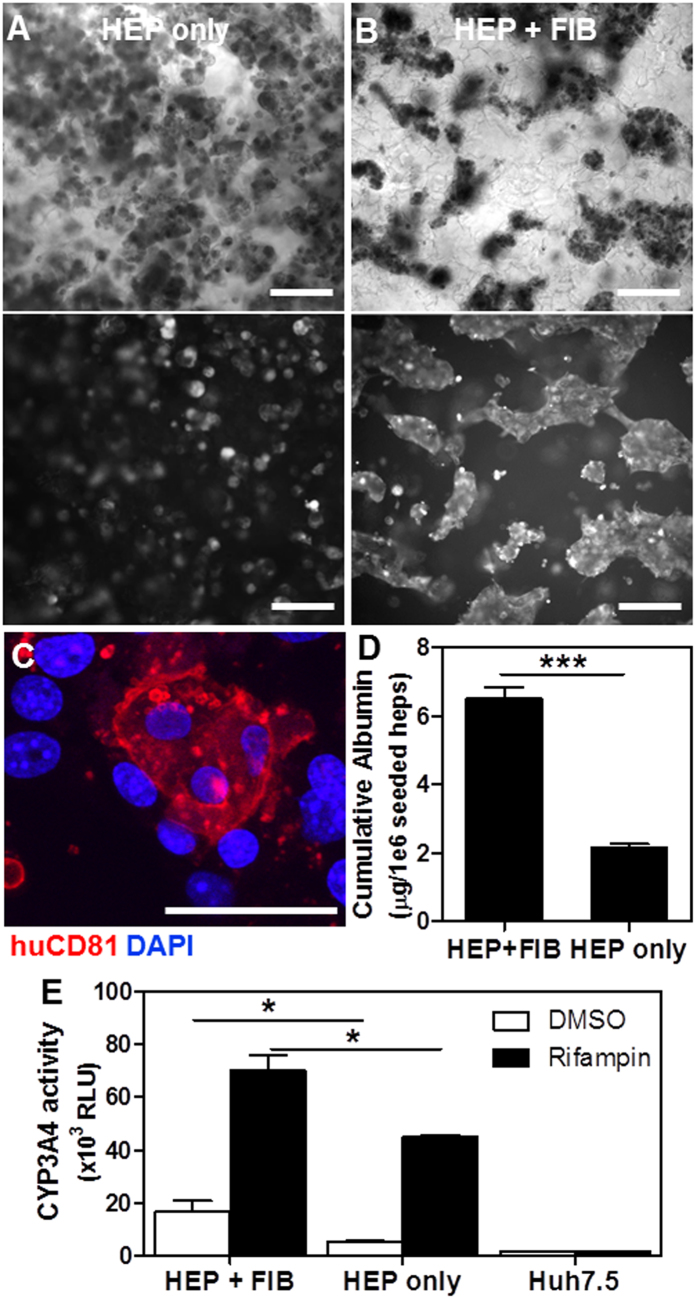
*In vitro* characterization of PEG cryogel‐based porous human ectopic artificial liver (p-HEAL). (**A**) Representative bright-field (top) and calcein fluorescence (bottom) images of a monoculture of primary human hepatocytes (HEPs) in PEG cryogels (HEP only). HEPs remain unicellular and do not exhibit any cellular aggregation. (**B**) Representative bright-field (top) and calcein fluorescence (bottom) image of cellular aggregates formed in a coculture of HEPs and 3T3-J2 fibroblasts (FIB) in PEG cryogels (HEP + FIB). (**C**) Representative multiphoton confocal image of a cellular aggregate containing HEPs that express cell-surface CD81 that are surrounded by CD81-negative FIBs. (**D**) Coculture in p-HEALs (HEP + FIB) increases cumulative human albumin secretion, as measured by ELISA every 2 days from days 1–15 post-seeding, compared to monoculture (HEP only). ***p < 0.001, two-tailed t-test. n = 4. (**E**) Coculture in p-HEALs (HEP + FIB) increases CYP3A4 activity in response to 3 days of rifampin treatment, compared to monoculture (HEP only). Huh7.5 hepatoma cells are used as a negative control for CYP3A4 activity. *p < 0.05, two-tailed t-test. n = 3. Error bars represent SEM. Scale bars: 50 μm.

Having resolved the accessibility of *Plasmodium* sporozoites to a cell-laden biomaterial, we next sought to assess primary human hepatocyte (HEP) survival and function in this setting. HEPs seeded onto lyophilized PEG cryogels alone remained largely unicellular with limited cellular aggregation (Fig. 2A). In contrast, in the presence of supporting cells, 3T3-J2 mouse embryonic fibroblasts (FIB), shown previously to phenotypically rescue HEPs in both two-dimensional (2D)^34^ and 3D *in vitro* hepatic cultures^22^, HEP+FIB cocultures in PEG cryogels formed cellular aggregates that are size-limited by the cryogel pores (Fig. 2B). These HEP+FIB aggregates maintained hepatocyte surface expression of CD81 (Fig. 2C), a tetraspanin that has been implicated as a host entry factor for *Plasmodium* sporozoites^35^. Importantly, HEP+FIB coculture was essential for maintaining HEP albumin secretion, a proxy for HEP function, for 15 days *in vitro* (Fig. 2D), and better supported the maintenance of CYP3A4 drug metabolism activity compared to monocultures of HEPs (Fig. 2E). These data suggest that porous human ectopic artificial livers (p-HEALs) comprising 3D HEP+FIB cocultures in PEG cryogels successfully maintain HEP survival and function *in vitro*.

### p-HEALs support rodent *Plasmodium* liver stage infection *in vitro*

**Figure 3:**
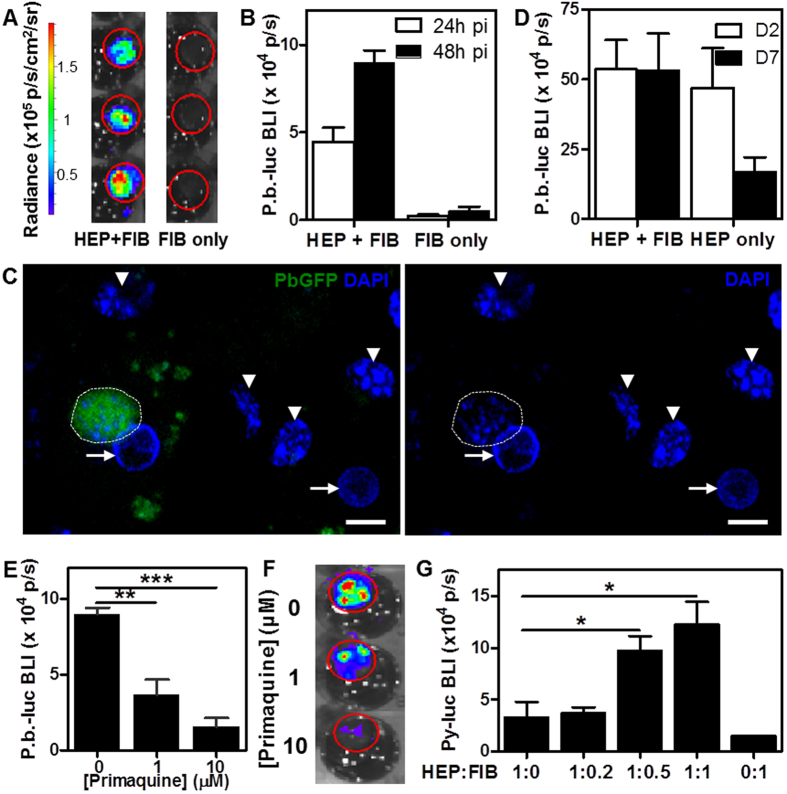
Establishment of liver stage malaria infection by two rodent *Plasmodium* species in p-HEALs *in vitro.* (**A**) Hepatocyte‐specific infection of p‐HEALs with Pb‐GFP-luc *in vitro*. PEG cryogels seeded either with HEP + FIB (p-HEAL) or FIB only (control) were exposed to Pb‐GFP-luc spz and imaged by bioluminescence imaging (BLI) at 48 h post-infection (3 wells per condition are shown). Each red circle demarcates the region of interest (ROI) that is defined for the quantification of the Pb-GFP-luc-derived BLI signal, as determined by the physical extent of a PEG cryogel. (**B**) BLI quantification of Pb‐GFP-luc infection in p-HEALs over time. n = 3. (**C**) Representative confocal images of Pb-GFP-luc (green) EEFs in p‐HEALs at 48 h post-infection. DAPI nuclear staining (blue) is shown either alone (right) or overlayed with GFP (left). A GFP-positive EEF (white dotted line) is observed adjacent to a HEP nucleus (white arrow). Arrowheads denote FIB nuclei. (**D**) Coculture of HEPs with FIBs maintains hepatocyte infectibility with Pb‐GFP-luc over time better than monoculture. PEG cryogels were seeded with HEP + FIB cocultures or HEP only monocultures and exposed to Pb-GFP-luc spz at 2 or 7 days after cell seeding. BLI quantification of Pb‐GFP-luc infection at 48 h post-infection is shown here. n = 3. (**E**) Dose response of Pb-GFP-luc-infected p-HEALs to antimalarial primaquine at 48 h post-infection. Primaquine treatment started at 3 h post-infection. **p < 0.01, ***p < 0.001, One way ANOVA (p = 0.0009) with Tukey’s multiple comparison test. n = 3. (**F**) Representative BLI images of Pb-GFP-luc-infected p-HEALs treated with various primaquine doses at 48 h post-infection (only 1 well per primaquine dose is shown here). Red circles demarcate the ROI that was quantified in (**E**). (**G**) Contribution of FIB density-driven heterotypic HEP/FIB interactions on infectibility of p-HEALs with an alternate *Plasmodium* species. At 2 days post-seeding, PEG cryogels seeded with varying HEP:FIB ratios were exposed to Py-luc spz, which is more restrictive in its host cell targeting, and imaged by BLI at 48 h post-infection. *p < 0.05, One way ANOVA (p = 0.0098) with Tukey’s multiple comparison test. n = 3. Error bars represent SEM. Scale bars: 10 μm.

Having functionally stabilized HEPs in PEG cryogels, the next goal was to determine whether p-HEALs were susceptible to *Plasmodium* sporozoite infection *in vitro.* p-HEALs were first exposed to Pb-GFP-luc sporozoites 2 days post-seeding and imaged by BLI to read out liver stage malaria infection. BLI signals were observed in p-HEALs (HEP+FIB) at 24 h and 48 h post-exposure to Pb-GFP-luc, but not in PEG cryogels seeded with an equivalent total number of FIBs alone (Fig. 3A,B). To confirm that Pb-GFP-luc EEFs were localized in HEPs and not FIBs, infected p-HEALs were fixed and immunostained for GFP expression. Pb-GFP-luc EEFs were observed primarily in cells containing a nucleus characteristic of HEPs (Fig. 3C), and predominantly in cells that express human CD81 (Figure S1A) or human arginase 1 (Figure S1B), consistent with earlier BLI measurements (Fig. 3A,B). While a small fraction (<5%) of Pb EEFs were detected in CD81-negative cells with fibroblast-like nuclear morphologies, non-hepatic tropism of rodent malaria sporozoites is well-documented^36^,^37^,^38^,^39^, and has been estimated to minimally contribute (~2%) to the pre-erythrocytic parasite load in *in vivo* rodent malaria models^39^. Furthermore, expression of the late liver stage marker, PbMSP-1, increased dramatically in Pb EEFs by 67 h post-infection, as compared with earlier time points (Figure S1C).

PEG cryogels seeded with only HEPs supported similar levels of infection as p-HEALs when infected with Pb-GFP-luc sporozoites at an early time-point, 2 days post-seeding (Fig. 3D), but exhibit decreased infectibility compared to p-HEALs if infection was not initiated until 7 days post-seeding, as determined by BLI levels 48 h post-infection (Fig. 3D). This observation is consistent with the finding that albumin secretion is maintained when HEPs are cocultured with FIB stromal cells (Fig. 2D) and suggests that functional activity of HEPs in p-HEALs correlates with the maintenance of HEP infectibility. To illustrate the potential utility of p-HEALs as a model for antimalarial drug testing, Pb-GFP-luc-infected p-HEALs were dosed with the antimalarial, primaquine, starting at 3 h post-infection. Pb-GFP-luc BLI signals measured at 48 h post-infection decreased in a dose-dependent manner with increasing primaquine concentrations (Fig. 3E,F). Given that primaquine requires bioactivation by hepatic drug metabolism enzymes (DME) to exert its antimalarial activity^40^,^41^,^42^, this finding is consistent with the earlier observation that HEPs in p-HEALs maintain their DME activity (Fig. 2E).

We next tested whether p-HEALs were infectible with an alternate rodent malaria parasite species, *P. yoelii* (Py), since Py exhibits more restricted host targeting than Pb and thus may better predict infectibility with the human malaria species *P. falciparum*^43^. p-HEALs were exposed to luciferase-expressing P. yoelii (Py-luc) sporozoites at 2 days post-seeding. Variation of HEP:FIB seeding ratios confirmed that Py-luc infection depended on the inclusion of FIB cells, and increased with higher FIB densities (Fig. 3G).

### p-HEALs support rodent *Plasmodium* liver stage infection *in vivo*

**Figure 4:**
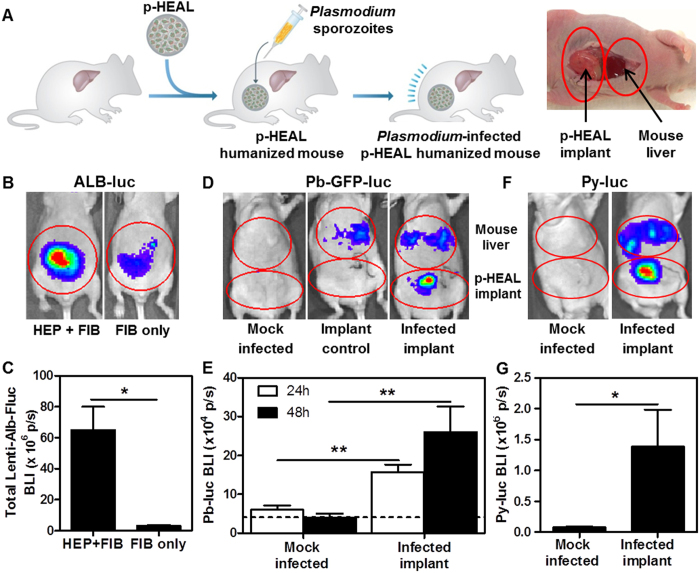
Characterization of *in vivo* p-HEAL implant survival and rodent *Plasmodium* liver stage infection *in vivo.* (**A**) (left) Schematic of humanization of mice with p-HEALs and liver stage *Plasmodium* infection. (right) Schematic of non-overlapping regions of interest for BLI measurements of Pb-GFP-luc or Py-luc infection in either the mouse liver or the implanted p-HEAL in p-HEAL-humanized mice after intragraft injection with either Pb-GFP-luc or Py-luc spz. (**B**) Representative BLI image of nude mice that received p-HEALs (HEP + FIB) or PEG cryogels with FIB only at D12 post-implantation. Prior to encapsulation, HEPs were transduced with a human albumin-driven lentiviral firefly luciferase (Lenti-Alb-Fluc) reporter. (**C**) Cumulative BLI signal from Lenti-Alb-Fluc reporter in mice shown in (**B**) over the course of 3 weeks post-implantation *in vivo*. BLI signals were measured every 2 or 3 days. *p < 0.05, two-tailed t-test. n = 3. (**D**,**E**) p-HEALs humanized mice were injected with 1.5 × 10^5^–5 × 10^5^ Pb-GFP-luc spz D5 post-implantation, and liver stage infection was measured by BLI at 24 h and 48 h post-infection. The dashed line (—) represents the background BLI signal from the implant site of mice that received acellular PEG cryogel implants and were subsequently injected with infectious Pb-GFP-luc sporozoites (Implant control). **p < 0.01, Mann-Whitney test. n = 5, 6, 17, 17 for mock infected 24 h, mock infected 48 h, infected implant 24 h and infected implant 48 h, respectively; n is cumulative from 4 experiments performed on separate days. (**F**,**G**) p-HEALs humanized mice were infected with 9 × 10^5^ Py-luc spz D5 post-implantation, and infection was measured by BLI at 48 h post-infection. *p < 0.05, Mann-Whitney test. n = 3, 7 for mock infected and infected implant, respectively. Error bars represent SEM.

After establishing the infectibility of p-HEALs and their drug responsiveness *in vitro*, we proceeded to assess whether p-HEALs offer the potential to serve as a humanized mouse model suitable for future preclinical studies of liver stage malaria infection (Fig. 4A). p-HEALs were transduced with a lentivirus reporter that expresses firefly luciferase under the control of the human albumin promoter (Figure S2A), and exhibited HEP+FIB co-culture dependent reporter expression *in vitro* for at least 13 days after transduction (Figure S2B). Transduced p-HEALs were then implanted in the IP space in athymic nude mice^22^ to determine whether p-HEALs can be functionally maintained *in vivo.* Whole-animal BLI showed that the IP implantation site supported HEP albumin promoter activity for 2–3 weeks post-implantation (Figs 4B,C, S2C), which suggests that p-HEALs represent a ‘next generation’ tissue engineered liver humanized mouse model, consistent with the ‘first generation’ PEG-based HEALs^22^.

To assess whether p-HEAL humanized mice could model liver stage malaria *in vivo*, established p-HEAL implants were initially challenged with Pb-GFP-luc sporozoites via intragraft injections and imaged 24 h and 48 h post-injection (Figure S2D). The IP location of the implanted p-HEALs did not overlap with the mouse liver and thus allowed for the demarcation of non-overlapping regions of interest for BLI measurements (Fig. 4D). The physical separation of p-HEALs and the mouse liver was necessitated by the potential for injected rodent-tropic Pb-GFP-luc sporozoites to infect the mouse liver via the circulation due to incomplete intragraft delivery, or leakage of sporozoites from the porous p-HEAL implant into the IP space. Indeed, a scattered, above background BLI signal was observed at the expected location of endogenous mouse liver in mice injected with Pb-GFP-luc sporozoites, relative to mock infected control animals (Fig. 4D). In 94% of p-HEAL-implanted mice injected with Pb-GFP-luc sporozoites (16/17 mice from 4 experiments), significantly higher BLI signals were detected at the site of the implanted p-HEAL, relative to background levels in mock-infected mice (Fig. 4D,E), suggesting that the implanted p-HEALs were infected with Pb-GFP-luc. As an additional control for the p-HEAL specificity of the BLI signal from the implant site, intragraft Pb-GFP-luc sporozoite injections in mice implanted with acellular PEG cryogels only elicited BLI signals that overlap with the mouse liver, but not the implant site (Fig. 4D,E). These findings extended to the more restricted rodent *Plasmodium* species, Py, as 57% of mice tested (4/7 mice in 1 experiment) injected with Py-luc spz exhibited specific, implant-localized BLI signals that were elevated relative to those observed in mock-infected animals (Fig. 4F,G), suggesting that the implanted p-HEALs were also infectible with Py-luc.

### Establishment of human *Plasmodium falciparum* liver stage infection in p-HEALs *in vitro* and *in vivo*

**Figure 5:**
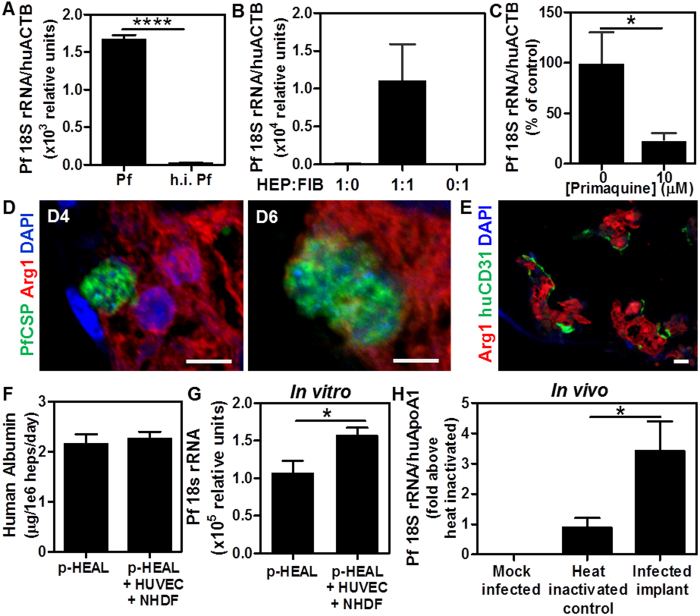
Establishment of human *Plasmodium falciparum* liver stage infection in p-HEALs *in vitro* and *in vivo.* (**A**) Infection of p-HEALs with Pf at D4 post-infection, as quantified via RT-PCR analysis of Pf18S rRNA levels, and validated with a heat-inactivated Pf spz control. ****p < 0.0001, two-tailed t-test. n = 3. (**B**) HEP+FIB coculture in p-HEALs is necessary for maintaining Pf infectibility of HEPs. (**C**) Treatment of Pf-infected p-HEALs with primaquine abrogates Pf infection in p-HEALs. *p < 0.05, two-tailed t-test. n = 9. (**D**) Representative confocal image of PfEEF in p-HEALs at D4 and D6 post-infection. HEP is immunostained for arginase 1 (Arg1), PfEEF is immunostained for Pf circumsporozoite protein (PfCSP). On average, one PfEEF was observed in every 5 sections of the 11 mm × 0.5 mm p-HEAL. Therefore, there are an estimated 4 PfEEFs per cm^2^ of p-HEAL tissue, based on observations of 5 μm transverse sections. (**E**) Representative confocal image of p-HEALs containing HUVECs and NHDFs in addition to HEPs and FIBs. HUVECs are immunostained for huCD31. (**F**) Effect of HUVEC and NHDF addition to p-HEALs on human albumin production by HEPs at D5 post-seeding *in vitro*. (**G**) Effect of HUVEC and NHDF addition to p-HEALs on Pf infection *in vitro* at D4 post-infection. *p < 0.05, Mann-Whitney test. n = 10. (**H**) p-HEALs containing HUVEC and NHDF were implanted into mice, and p-HEAL humanized mice were injected with 1–1.2 × 10^6^ Pf spz at D4 post-implantation. Infection was measured by RT-PCR on explanted p-HEALs at D3.5 post-infection. Data is normalized to implants that received heat-inactivated spz. *p < 0.05, Mann-Whitney test. n = 3, 9, 10 for mock infected, heat inactivated and infected implant, respectively; n is cumulative from 2 experiments performed on separate days, with a take-rate of 60% (6/10 mice had Pf18S rRNA levels above the heat-inactivated control). Error bars represent SEM. Scale bars: 10 μm.

We next sought to test whether p-HEALs optimized to be infectible by rodent malaria species would also be infectible by the human malaria species, *P. falciparum* (Pf). In the absence of an engineered strain of Pf expressing a luciferized reporter, p-HEALs that were exposed to Pf sporozoites *in vitro* at 2 days post-seeding were assayed for infection by RT-PCR amplification of Pf 18S ribosomal RNA (rRNA). Positive Pf 18S rRNA levels were detected in p-HEALs at 4 days post-exposure, but not in p-HEALs exposed to heat-inactivated sporozoites (Fig. 5A) or PEG cryogels containing only FIBs (Fig. 5B). HEP+FIB coculture was necessary to maintain infectibility with Pf (Fig. 5B), and primaquine treatment of p-HEALs starting at 3 h post-infection abrogated Pf 18S rRNA levels in Pf-infected p-HEALs (Fig. 5C), consistent with the findings obtained following infection with rodent *Plasmodium* species. Pf EEFs were also visually confirmed to be localized within HEPs at both 4 and 6 days post-infection via immunostaining assays using reagents that detect a Pf antigen (PfCSP) and a HEP antigen (Arg1) (Fig. 5D).

To determine whether the infectibility of p-HEAL humanized mice by rodent *Plasmodium* species can be extended to the human *Plasmodium* species, p-HEAL humanized mice were challenged with intragraft injections of Pf sporozoites and explanted 3–5 days later for RT-PCR analysis of Pf 18S rRNA (Figure S2D). While rodent *Plasmodium* sporozoites productively infected p-HEAL humanized mice (Fig. 4E–H), pilot *in vivo* Pf studies revealed only low levels of Pf 18S rRNA in implanted p-HEALs injected with either native or heat-inactivated Pf sporozoites (Figure S3), suggesting that the *in vitro* infectibility of p-HEALs by Pf was not maintained *in vivo*. To improve Pf infection *in vivo*, we hypothesized that the inclusion of endothelial cells and supporting mural cells could provide additional paracrine factors that better maintain HEP survival or their ability to support Pf infection *in vivo*^44^,^45^,^46^. The seeding of human umbilical venous endothelial cells (HUVECs) and normal human dermal fibroblasts (NHDFs) into p-HEALs resulted in the formation of human CD31-positive vessel-like structures around HEP aggregates *in vitro* (Fig. 5E), similar to the self-organizational behavior of endothelial cells previously observed in other engineered tissues *in vitro*^47^,^48^. The additional supportive cell populations in p-HEALs yielded no enhancement in HEP albumin production (Fig. 5F), but nonetheless mediated a small but significant increase in p-HEAL infectibility by Pf *in vitro* (Fig. 5G), supporting the hypothesis that alternate paracrine signals may promote HEP susceptibility to Pf. When this strategy was applied to generate humanized mice bearing p-HEALs with HUVECs and NHDFs, 60% of the mice challenged with intragraft injection of Pf sporozoites exhibited Pf 18S rRNA levels that exceeded those observed in mice receiving heat-inactivated Pf sporozoites, by an average of 3-fold (6/10 mice in 2 experiments) (Fig. 5H). Taken together, these data demonstrate that the ectopic implantation of p-HEALs into uninjured hosts can generate humanized mice that support productive liver stage human malaria infection.

## Discussion

In this study, we designed an implantable PEG-based human ectopic artificial liver (HEAL) that is amenable to liver stage *Plasmodium* infection *in vitro* and *in vivo*. By introducing macroporosity into PEG via cryogelation, we created macroporous PEG cryogels that allow *Plasmodium* sporozoites to access the hepatocytes encapsulated within. Furthermore, by introducing appropriate densities of both primary human hepatocytes and stromal fibroblasts, we obtained porous HEALs (p-HEALs) that support hepatic functional maintenance, and more importantly, infectibility with liver stage *Plasmodium* sporozoites *in vitro*. Finally, we demonstrated that p-HEALs survive intraperitoneal implantation and support infection with both liver stage rodent and human *Plasmodium* parasites *in vivo*.

Many *in vitro* models that recapitulate liver stages of human malaria in its native host cell, the primary human hepatocyte, have been described^3^,^49^,^50^. However, inquiries regarding various pharmacokinetic, pharmacodynamic, toxicological, immunological and pathophysiological factors tend to involve extra-hepatic components of the *in vivo* microenvironment, and thus necessitate the development of *in vivo* liver stage malaria models. Humanized mouse models of liver stage malaria potentially better reflect the physiology of both the human host cell and the human parasite compared to existing rodent models^10^, and to date, genetic liver injury models have been successfully shown to support liver stage human malaria^17^,^19^,^20^. While genetic liver injury models provide a robust tool for the study of liver stage human malaria, their relatively high cost may limit their application in large cohort studies for preclinical antimalarial drug development, despite their commercial availability. The development of mice humanized with tissue-engineered HEALs has enabled rapid humanization of healthy mice with normal liver function in a facile, more scalable and economical fashion^22^. The adaptation of the HEALs platform for modeling liver stage malaria *in vivo* is a potential way to establish a more accessible model that could be more easily scaled up for large preclinical antimalarial drug studies. Although p-HEALs containing only HEP and FIB support infection with rodent malaria sporozoites (Pb-GFP-luc or Py-luc) *in vivo*, their infectibility with human malaria sporozoites (Pf) *in vivo* appears to be dependent on their co-encapsulation with HUVEC and NHDF (Figs 5H, S3). This apparent dependence on additional endothelial-derived paracrine factors could reflect a more stringent hepatic microenvironmental requirement for Pf to productively infect HEPs compared with rodent malaria species, which is partially supported by the comparatively more restricted infectibility of HEPs by Pf than either Pb or Py – a trend that has been previously observed *in vitro*^3^,^31^. Furthermore, *Plasmodium* sporozoites have been observed to traverse endothelial cells *in vitro* and *in vivo*^51^, and may experience a hypothetical activating signal following contact with human endothelial cells that promotes host cell invasion. Although the inclusion of HUVEC and NHDF enabled p-HEAL humanized mice to support Pf infection *in vivo*, the current 60% Pf take-rate and the lack of gene expression of mid to late liver stage antigens such as MSP-1 or EBA-175 by Pf EEFs (data not shown) suggests that the hepatic microenvironment in p-HEALs requires further optimization to improve the reproducibility of Pf infection, as well as to augment parasite development post-infection. For example, the current use of non-liver derived HUVEC could contribute to a lack of liver-specific paracrine interactions between HEPs and liver sinusoidal endothelial cells (LSECs) that typically involve survival factors such as VEGF (from HEPs) and HGF or IL-6 (from LSECs)^52^. The resultant lack of HGF might indirectly inhibit EEF maturation, given the critical role that HGF plays in liver stage malaria infection and progression^53^. The immunological environment in p-HEALs is another potentially important parameter, given that liver stage malaria infection induces a robust type I interferon (IFN) response which inhibits EEF development, and IFN-stimulated genes are primarily expressed by HEPs^54^. Although this inhibitory effect appears to depend on liver leukocytes^54^, exogenous inhibition of the type I IFN response in Pf-infected primary human hepatocytes increased liver stage Pf infection levels and EEF development *in vitro* (unpublished observations), suggesting that the innate hepatocyte immune response to an active liver stage infection might suffice to limit EEF maturation in p-HEALs. The absence of liver-specific non-parenchymal cells like Kupffer cells and LSECs could compound this situation, since these cells produce anti-inflammatory cytokines such as IL-10, which help to maintain a tolerogenic hepatic microenvironment *in vivo*^55^.

The development of p-HEALs as a porous biomaterial-based engineered artificial human liver with a tailorable hepatic microenvironment that can be infected with liver stage malaria both *in vitro* and *in vivo* offers a unique comparative test bed to uncover specific microenvironmental parameters that influence human hepatocyte infectibility with malaria. No other 3D hepatic model currently offers this capacity without being subject to confounding variables that differ between the paired *in vitro* and *in vivo* systems chosen for the comparison. Among the various *in vitro* liver stage malaria models or 3D human liver models that exist, most cannot be readily translated to an *in vivo* setting. For example, models that involve 2D or 3D cultures in multiwell plates or bioreactors that depend on intricate microfluidics systems preclude *in vivo* transplantation^56^,^57^. Many biomaterial-based 3D human liver systems may preclude *in vitro* liver stage malaria infection due to a mismatch between their material nanoporosity and the micron-sized dimensions of *Plasmodium* sporozoites. Another advantage of the p-HEAL platform is that its design features enable it to be rapidly generated (on the order of days) and available for immediate use (less than a week post-engraftment) in short-term infection and drug/vaccine efficacy assays, as opposed to other platforms designed for regenerative and/or biological studies that would extend for months after transplantation. We anticipate that next-generation p-HEALS will incorporate updated design structures so as to become suitable for studies of long-term infections, such as the liver stages of *P. vivax*.

For the purposes of disease modeling and preclinical testing, it would be advantageous to introduce a human immune system (HIS) component to the p-HEAL model. The resulting ‘dual humanized’ mouse would thereby allow for modeling both liver and blood stages of human malaria in a small animal^11^. Such a model would enable the study of host-parasite interactions in the context of a human-specific adaptive immune response, and more practically could be useful as a small animal challenge model for pre-erythrocytic vaccine candidates^58^,^59^,^60^. The DRAG and TK-NOG models are the first such dual humanized mouse models reported to sustain the complete development of both liver and blood stages of *P. falciparum* and/or *P. ovale in vivo*^21^,^61^, but like other genetic liver injury models may be practically difficult to scale up to larger cohort sizes. To achieve co-engraftment of HEALs and HIS, it would be essential to promote stable vascularization in p-HEALs, so as to maximize circulation-mediated interactions between the humanized components. p-HEAL vascularization could be achieved via tissue engineering strategies including pre-vascularization of engineered tissue that is driven by cellular self-organization^26^,^47^,^48^, or micropatterning^62^ of angiogenic and vasculogenic cell types prior to transplantation, microfabrication of vascular units with accompanying surgical anastomosis during implantation^63^,^64^, or incorporation of angiogenic factors like VEGF, bFGF and PDGF into engineered tissue^65^,^66^. To establish liver stage *Plasmodium* infection via a more physiologically relevant infection route like mosquito bites, the engineered vasculature in p-HEALs should mimic the microenvironment of a liver sinusoid so that *Plasmodium* sporozoites will preferentially sequester in and extravasate from these engineered vessels in the implanted tissue, like they naturally do in the liver sinusoid^67^,^68^, and subsequently invade the surrounding hepatocytes. Liver sinusoid-like structures have been created by cellular self-assembly *in vitro*^69^ or reseeding decellularized liver with primary human liver and endothelial cells^70^,^71^, and offer the potential for generating engineered vasculature in p-HEALs that are more liver-specific than achievable with other strategies.

In summary, this study provides a proof-of-concept that human engineered artificial livers can support liver stage malaria infection, and may have the potential to be applied to the development of humanized mouse models of liver stage human malaria in a facile, rapid, and scalable fashion for preclinical studies.

## Materials and Methods

### Cell culture and reagents

Cryopreserved primary human hepatocytes were purchased from vendors permitted to sell products derived from human organs procured in the United States by federally designated Organ Procurement Organizations. Vendors included CellzDirect (Lot Hu1434) and Celsis *In vitro* Technologies (Lot NON). Human hepatocyte culture medium was high glucose Dulbecco’s Modified Eagle’s Medium (DMEM) with 10% (v/v) fetal bovine serum (FBS), 1% (v/v) ITS^TM^ (BD Biosciences), 7 ng/ml glucagon, 40 ng/ml dexamethasone, 15 mM HEPES, and 1% (v/v) penicillin-streptomycin. FIB-3T3 murine embryonic fibroblasts (gift of Howard Green, Harvard Medical School) were cultured at <18 passages in fibroblast medium comprising of DMEM with high glucose, 10% (v/v) bovine serum, and 1% (v/v) penicillin-streptomycin. Polyethylene glycol diacrylate (Mn 3.4 kDa) was purchased from Laysan Bio Inc and used without further purification. Ammonium persulfate (APS), N,N,N′,N′-tetramethylethylenediamine (TEMED), and primaquine diphosphate were obtained from Sigma. Primaquine diphosphate was dissolved at 0.5 mg/mL in deionized water, and stored as single-use aliquots at −20 °C.

### Synthesis of PEG cryogels

PEG cryogels (PCs) were synthesized as previously described^29^. PEGDA was dissolved in sterile phosphate buffered saline (PBS) to a working concentration of 10% w/v and kept on ice. A final working concentration of the initiator–accelerator mixture comprising 0.5% w/v of ammonium persulfate (APS) and 0.05% w/v of N,N,N′,N′-tetramethylethylenediamine (TEMED) was added to the 10% w/v PEGDA solution. 75 μL or 150 μL of the reaction mixture were dispensed into 96- or 48-well plates respectively and polymerized at −20 °C for 20 h. After gelation, PCs were rehydrated with sterile water, sterilized with three changes of 70% ethanol, and then washed extensively with fresh sterile water to remove all unreacted compounds. PCs synthesized in 96- or 48-well plates swelled to the well dimensions of 48- and 24-well plates respectively and are labeled PC96 or PC48 respectively.

### Fabrication of p-HEALs

PCs were lyophilized overnight into a sponge-like scaffold. PC96s were used for *in vitro* experiments whereas PC48s were used for *in vivo* experiments. Primary human hepatocytes were mixed with FIB-3T3 mouse embryonic fibroblasts in a 1:1 ratio and seeded at 350,000 hepatocytes per PC96 sponge, and proportionately scaled up to 935,000 hepatocytes per PC48 sponge. The seeding volumes were 75 μL (PC96) and 150 μL (PC48). After cells were dispensed onto the lyophilized PCs, the constructs were centrifuged at 50 g for 3 min to encourage cellular infiltration into the pores of the PCs. The seeded p-HEALs were then incubated at 37 °C for 15 min, before more hepatocyte medium was added and a second centrifugation at 50 g for 3 min was carried out. After that, sufficient medium was added to barely cover the p-HEALs, and they were incubated under conventional growth conditions.

### Biochemical assays

Cell culture supernatants were collected and stored at −20 °C. Albumin content was measured by an enzyme-linked immunosorbent assay using goat anti-human albumin antibody (Bethyl Labs) with horseradish peroxidase detection (Bethyl Labs) and 3,3′,5,5′-tetramethylbenzidine (TMB, Pierce) development. For CYP450 induction studies, *in vitro* p-HEALs were treated daily for 3 days with CYP450 inducer rifampin (20 μM). Subsequently, CYP3A4 activity was measured using the P450-Glo™ CYP3A4 Assay with Luciferin-IPA (Promega) according to the manufacturer’s instructions.

### *Plasmodium* sporozoites

GFP- and luciferase-expressing P. berghei ANKA (Pb-GFP-luc) and luciferase-expressing *P. yoelii* (Py-luc) sporozoites were obtained by dissection of the salivary glands of infected Anopheles stephensi mosquitoes obtained from the insectaries at New York University (New York, New York, USA) or Harvard School of Public Health (Boston, Massachusetts, USA). *P. falciparum* (Pf) sporozoites were obtained by dissection of the salivary glands of infected Anopheles gambiae mosquitoes obtained from the insectary at Johns Hopkins School of Public Health (Baltimore, Maryland, USA).

### *Plasmodium* infection of p-HEALs *in vitro*

p-HEALs (PC96s) were transferred on to sterile gauze for about 20 seconds and then transferred to a clean and dry well of a new multiwall plate. The sterile gauze removes significant amounts of culture medium from the p-HEAL. *Plasmodium* sporozoites (4 × 10^4^–8 × 10^4^ Pb-GFP-luc, 1 × 10^5^ Py-luc or 1.5 × 10^5^–5 × 10^5^ *P. falciparum*) were diluted in culture medium with a final concentration of 3% v/v penicillin/streptomycin and 10% v/v fetal bovine serum and dispensed on to p-HEALs in volumes of approximately 50 μL, which saturates the cryogel but ideally does not overflow down the sides of the cryogel. The sporozoite solution was readily taken up by capillary action into the semi-dry p-HEALs, which were then incubated at 37 °C for 1 h to allow sporozoite invasion of hepatocytes without the addition of more culture medium (to avoid washing out the sporozoites from within the cryogel). Medium was added 1 h after infection to prevent the p-HEALs from drying out. 3 h after exposure to sporozoites, the p-HEALs were washed thrice with hepatocyte medium containing 3% v/v penicillin/streptomycin and incubated under growth conditions with hepatocyte medium containing 3% v/v penicillin/streptomycin and 0.1% v/v Fungizone^®^ antimycotic (Gibco^®^). To read out Pb-GFP-luc or Py-luc infection, an IVIS bioluminescence imaging system was used. To read out Pf infection, Pf-infected samples were lysed with TRIzol^®^ (Invitrogen) or RLT buffer (Qiagen) and processed for RT-PCR as described below.

### Whole mount immunohistochemistry (*P. berghei*)

All the following steps were performed on an orbital shaker. p-HEALs were washed twice with PBS for 10 min each, fixed with 4% paraformaldehyde for 30 min, washed thrice with PBS for 10 min each at room temperature, then permeabilized with 0.1% Triton X-100 in PBS for 10 min at 4 °C, and rinsed twice with PBS for 5 min each. The samples were blocked with 2% bovine serum albumin in PBS overnight at 4 °C, and then incubated with a primary antibody diluted in 2% BSA/0.05% Tween-20/PBS: mouse anti-human CD81 (clone JS-81, BD Pharmingen; 1:100), rabbit anti-GFP-AlexaFluor488 (Invitrogen, 1:100) overnight at 4 °C. The samples were washed thrice with 0.05% Tween-20/PBS PBS for 1–2 h each at 4 °C, then incubated with a secondary antibody diluted in 2% BSA/0.05% Tween-20/PBS: donkey anti-mouse-AlexaFluor594 (Invitrogen; 1:400) overnight at 4 °C. The samples were counterstained with Hoechst 33258 (Invitrogen; 1:1000) in PBS and washed thrice with PBS for 1–2 h each at 4 °C, and stored in PBS at 4 °C until imaging.

### Immunohistochemistry (*P. falciparum*)

At 3 or 6 days post-infection, p-HEALs were fixed in 4% paraformaldehyde for an hour at room temperature, processed and paraffin-embedded for immunohistochemistry. Prior to cell seeding, PEG cryogels have a diameter equivalent to that of a well of a 48-well plate (11 mm), and typically exhibit a depth of 0.5 mm. After cells seeding and infection, pHEAL constructs are sectioned transversely for use in immunohistochemical staining. Therefore, each 5 μm-p-HEAL section covers a cell-seeded area of approximately 5 mm^2^ (11 mm × 0.5 mm). Paraffin sections were double-stained with the following primary antibodies: mouse anti-PfCSP (clone 2A10, the purified antibody was obtained through the MR4 as part of the BEI Resources Repository, NIAID, NIH: Mus musculus (B cell); Mus musculus (myeloma) 2A10, MRA-183, deposited by E Nardin; 1:500) and rabbit anti-human arginase-1 (Sigma; 1:400) overnight at 4 °C. The sections were washed and stained with the following secondary antibodies: donkey anti-mouse-AlexaFluor488 (Invitrogen; 1:1000) and donkey anti-rabbit-AlexaFluor594 (Invitrogen; 1:1000), then counterstained with Hoechst 33258 and mounted with Fluoromount G (Southern Biotech). On average, one PfEEF was observed in every 5 sections. Therefore, there are an estimated 4 PfEEFs/cm^2^/5 μm-p-HEAL section.

### Microscopy

Immunostained samples were imaged on a Nikon Eclipse TE200, Olympus FV1000 Multiphoton Laser Scanning Confocal Microscope, or Olympus FV1200 Laser Scanning Confocal Microscope.

### Implantation and monitoring of p-HEALs

All animal experiments were approved by the Committee for Animal Care in the Department of Comparative Medicine at Massachusetts Institute of Technology. All animal experimental methods were carried out in accordance with the approved guidelines. p-HEALs were placed in the peritoneal cavity of anesthetized NCr nude mice (Taconic) following a 1-cm incision, and sutured to the mesenteric fat pads on both sides to prevent the p-HEALs from becoming obscured by the mouse organs in the intraperitoneal space. To noninvasively monitor transplanted p-HEALs for hepatocyte survival, HEALs were transduced with lentiviral pseudoparticles expressing firefly luciferase under the human albumin promoter (pTRIP.Alb.IVSb.IRES.tagRFP-DEST, 1:5 dilution; gift of Charles Rice, The Rockefeller University, New York) 3 days after fabrication and prior to implantation. Mice were injected with 250 uL of 15 mg/mL D-Luciferin (in PBS) (Caliper Life Sciences) in the intraperitoneal cavity and imaged using the IVIS Spectrum (Xenogen) system and bioluminescence was quantified using Living Image Software (Caliper Life Sciences).

### *Plasmodium* infection of p-HEALs mice

1.5 × 10^5^–5 × 10^5^ *P. berghei*-GFP-luc, 2.5 × 10^5^–1 × 10^6^ *P. yoelii*-GFP-luc or 5 × 10^5–1.5^ × 10^6^ *P. falciparum* sporozoites in 50–150 μL of DMEM were delivered into p-HEALs in p-HEAL mice by injecting directly into the implanted p-HEAL (intragraft injection). Intragraft injections were facilitated by implant boundaries that are visible through the mouse tissue.

### RNA isolation and RT-PCR

p-HEALs were explanted from mice 3–5 days after injection with *P. falciparum* sporozoites. The fat pad was detached from the p-HEAL, and any obvious fibrous capsule was removed. p-HEALs explants were stored in RNAlater (Ambion) until processed. Total RNA was isolated either using the TRIzol^®^ (Invitrogen) method or the RNeasy Mini Kit (Qiagen). p-HEALs were homogenized in TRIzol^®^ (Invitrogen) or RLT buffer (RNeasy Mini Kit, Qiagen) using mortar and pestle or the ‘RNA 02’ tissue homogenization protocol in a gentleMACS dissociator using gentleMACS M tubes (Miltenyi Biotec). For samples processed in TRIzol^®^, the lysates were used directly. For samples processed with the RNeasy Mini Kit, the lysates were first briefly centrifuged at 3500 rpm, transferred to 1.5 mL Eppendorf tubes, centrifuged at 14,000 rpm to pellet the cryogel debris and only the supernatants were used. cDNA was made using the iScript cDNA synthesis kit (Biorad) according to the manufacturer’s instructions. RT-PCR was carried out using the iScript one-step RT-PCR kit with SYBR^®^ Green (Biorad) in a CFX96™ Real-Time PCR Detection System (Biorad) according to the manufacturer’s instructions. The primers used to detect infections were *P. berghei* 18S rRNA (forward: GGAGATTGGTTTTGACGTTTATGTG, reverse: AAGCATTAAATAAAGCGAATACATCCTTAC) or *P. falciparum* 18S rRNA (forward: CTGGTTTGGGAAAAGCAAAT, reverse: CTCAATCATGACTACCCGTCTG), and endogenous controls for human beta actin (ACTB, Applied Biosystems) or human apolipoprotein A1 (forward: AGCGTGACCTCCACCTTCAG, reverse: CCTTCACCTCCTCCAGATCCTT) were used for housekeeping purposes.

### Statistics

Experiments were repeated three or more times with duplicate, triplicate or more samples for each condition, unless otherwise stated. Data from representative experiments are presented and similar trends were seen in multiple experiments, unless otherwise stated. Two-tailed t-tests or Mann-Whitney tests were performed for comparisons involving two conditions and one way ANOVAs were performed for comparisons involving three or more conditions with Tukey’s post-hoc test for multiple comparisons. All error bars represent s.e.m.

## Additional Information

**How to cite this article:** Ng, S. *et al*. Towards a Humanized Mouse Model of Liver Stage Malaria Using Ectopic Artificial Livers. *Sci. Rep.* **7**, 45424; doi: 10.1038/srep45424 (2017).

**Publisher's note:** Springer Nature remains neutral with regard to jurisdictional claims in published maps and institutional affiliations.

## Supplementary Material

Supplementary Information
