# Supplementary material for: Towards a Humanized Mouse Model of Liver Stage Malaria Using Ectopic Artificial Livers
Source: Sci Rep. 2017 Mar 31;7:45424. doi: 10.1038/srep45424 (PMC5374446; doi:10.1038/srep45424)
Supplement: Supplementary Information [file srep45424-s1.pdf]

Supplementary information

SREP-15-02660B

Towards a Humanized Mouse Model of Liver Stage Malaria Using Ectopic Artificial Livers

Shengyong Ng, Sandra March, Ani Galstian, Nil Gural, Kelly R. Stevens, Maria M. Mota, Sangeeta N. Bhatia

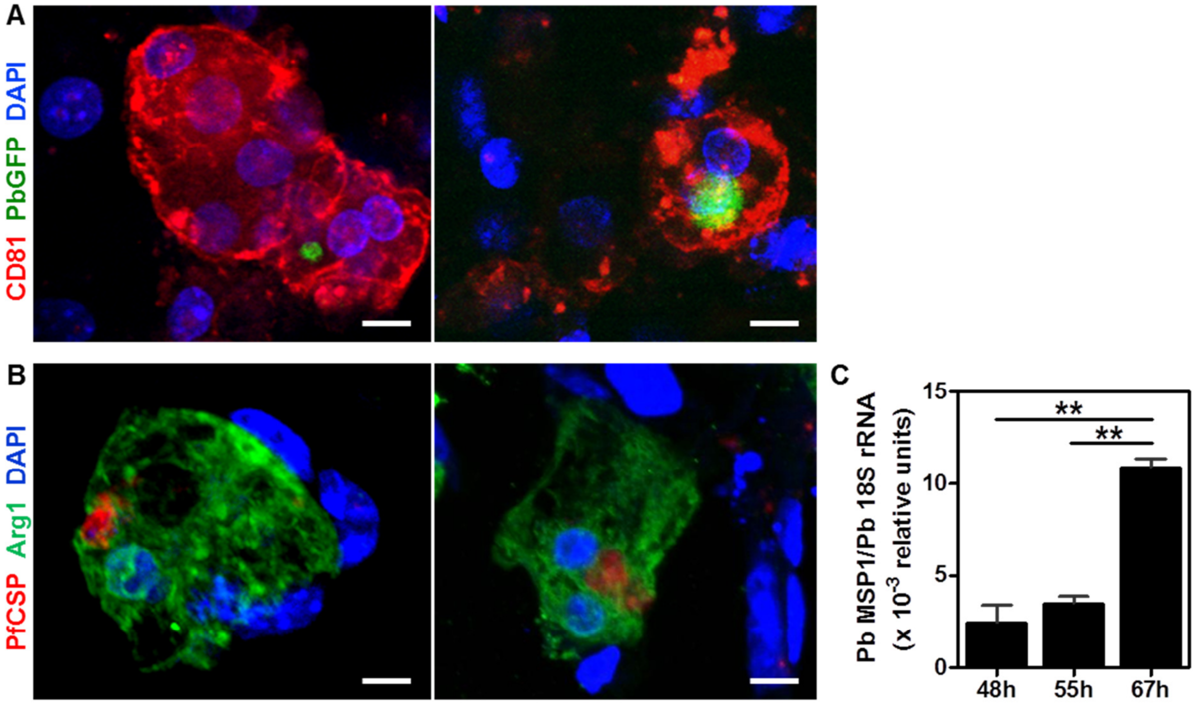

Figure S1. ***Plasmodium*** **liver stage infection of HEPs in p-HEALs.** (A) Representative confocal images of GFP- and luciferase-expressing *P. berghei* (Pb-GFP-luc) EEFs in PC-HEALs at 48h post-infection, with HEPs counterstained for human CD81 (red). (B) Representative confocal image of a Pf EEF in PC-HEAL at D3.5 post-infection, with HEPs counterstained for arginase 1 (Arg1, green). Scale bars: 10μm. (C) Maturation of Pb EEFs in p-HEALs infected with Pb, as quantified via RT-PCR analysis of PbMSP1 rRNA levels that have been normalized relative to a Pb 18S rRNA housekeeping gene.

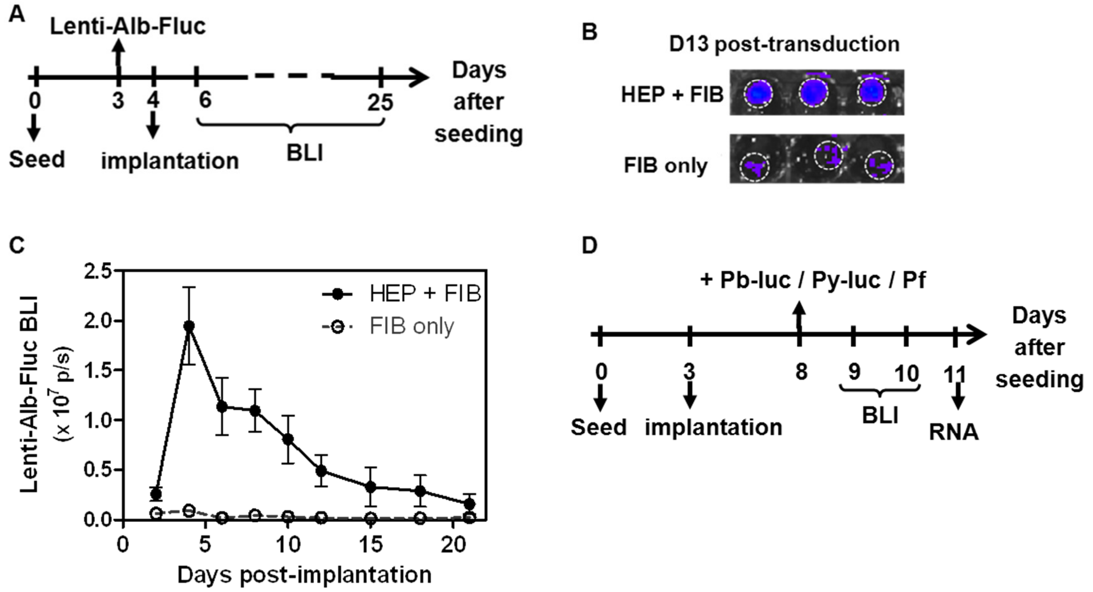

Figure S2. Characterization of lentiviral transduction of p-HEALs. (A) Experimental timeline for the lentiviral transduction of p-HEALs with lenti-Alb-Fluc reporter, implantation in nude mice, and non-invasive monitoring of implant survival post-implantation via bioluminescence imaging (BLI). (B) p-HEALs (HEP + FIB) or FIB only constructs, transduced with Lenti-Alb-Fluc reporter, and assayed for BLI signals at D13 post-transduction *in vitro*. (C) *In vivo* kinetics of lenti-Alb-Fluc reporter activity in p-HEAL or FIB only implants. (D) Experimental timeline for the implantation of p-HEALs, infection with *Plasmodium* sporozoites, and post-infection detection of infection by either bioluminescence imaging (Pb-GFP-luc or Py-luc) or explant analysis with RT-PCR (Pf).

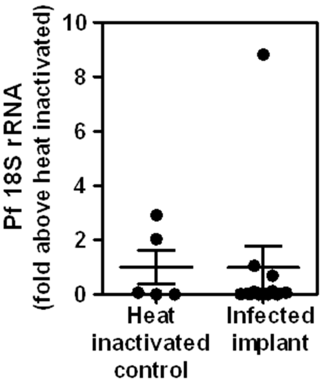

Figure S3. ***In vivo P. falciparum*** **infection of p-HEALs without HUVEC and NHDF.** p-HEALs (without HUVEC and NHDF) were implanted into mice, and humanized mice were infected with Pf at D4 post-implantation. Infection levels were measured at D3.5 post-infection by RT-PCR. n = 5, 11 for heat inactivated control and infected implant, respectively, n is cumulative from 2 experiments performed on separate days.
